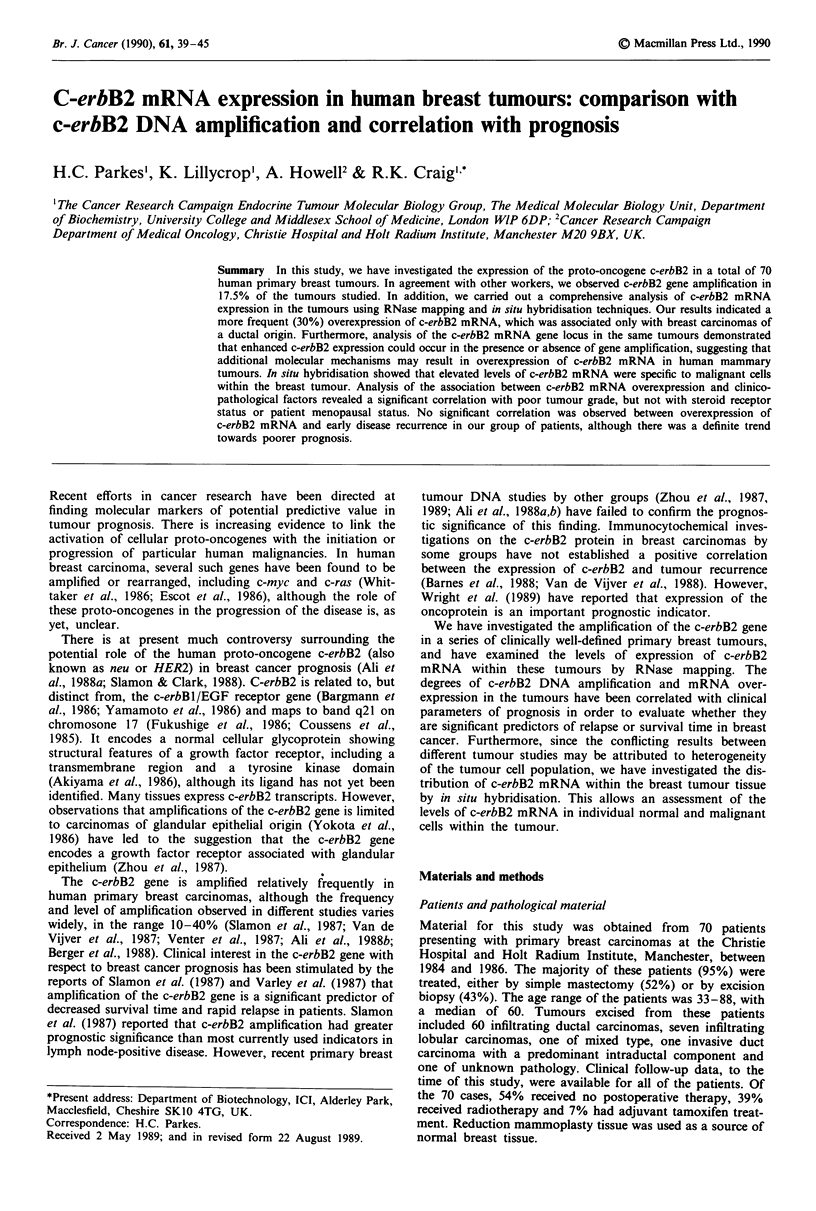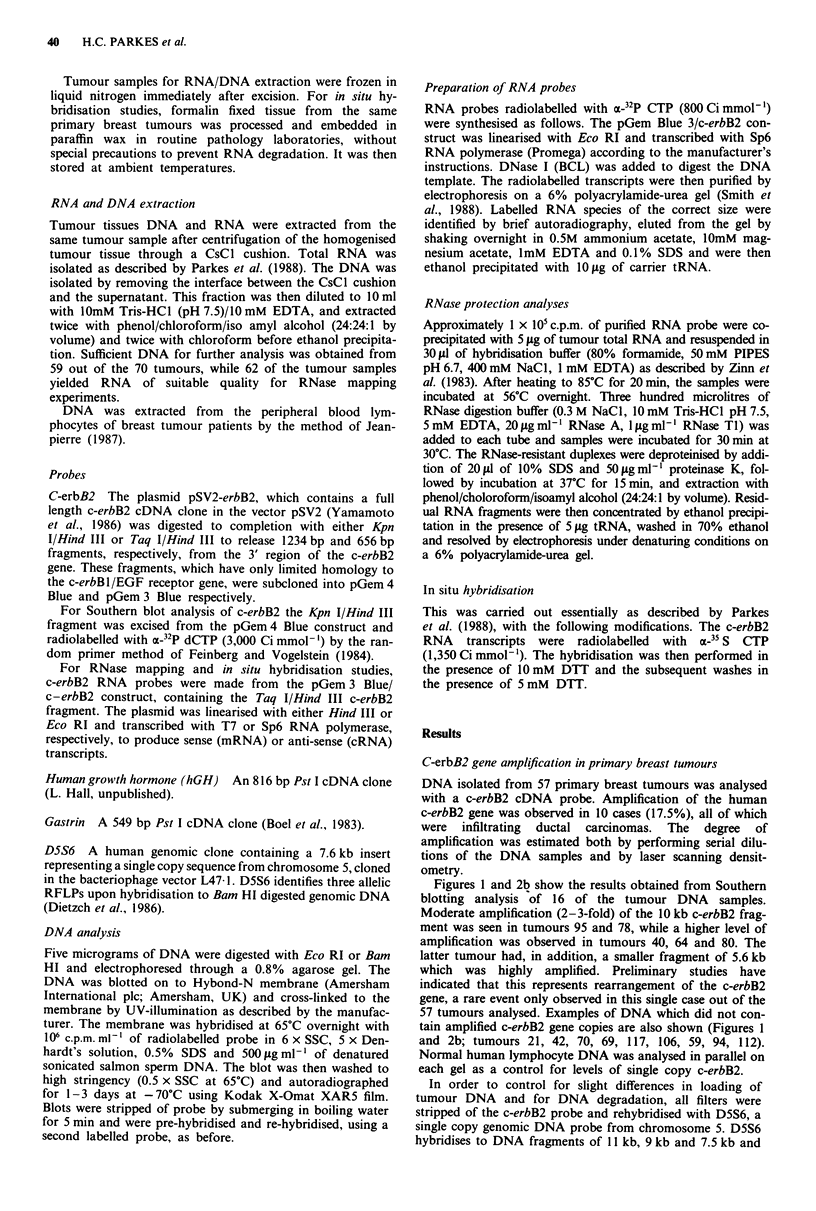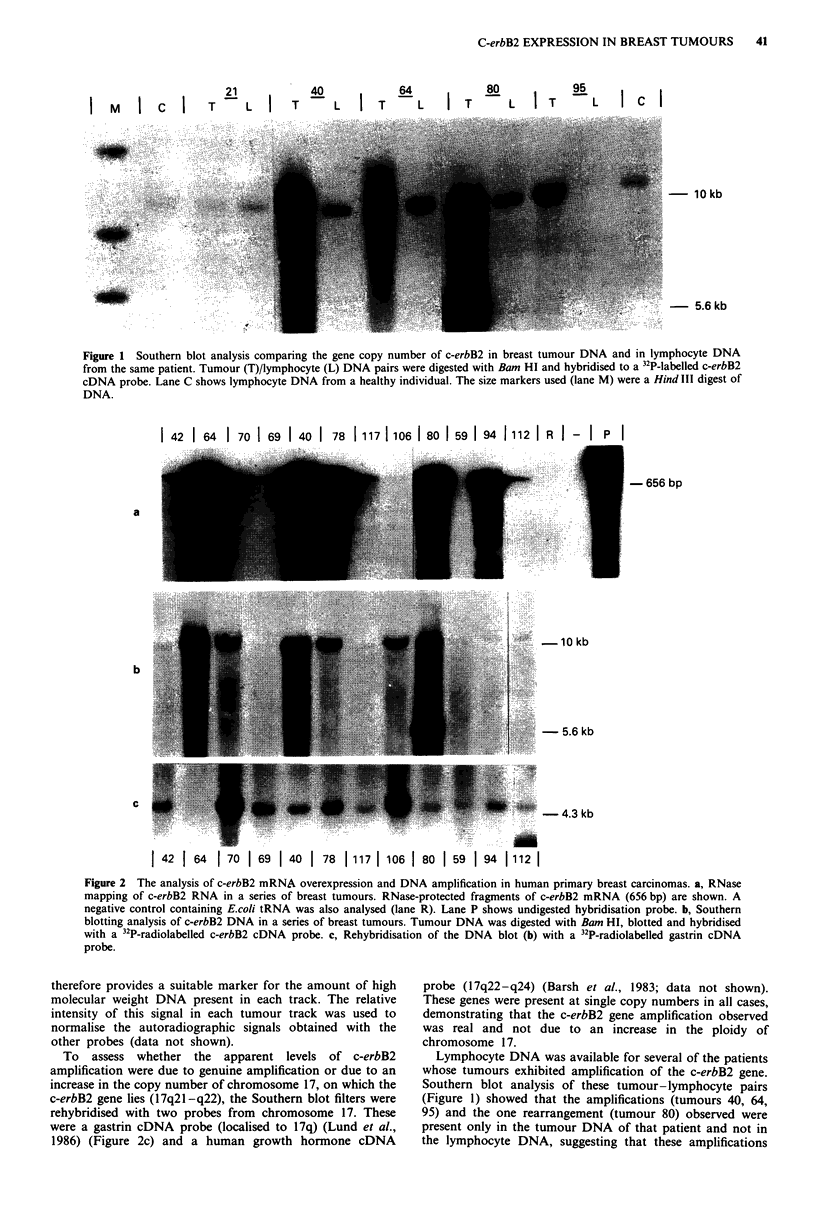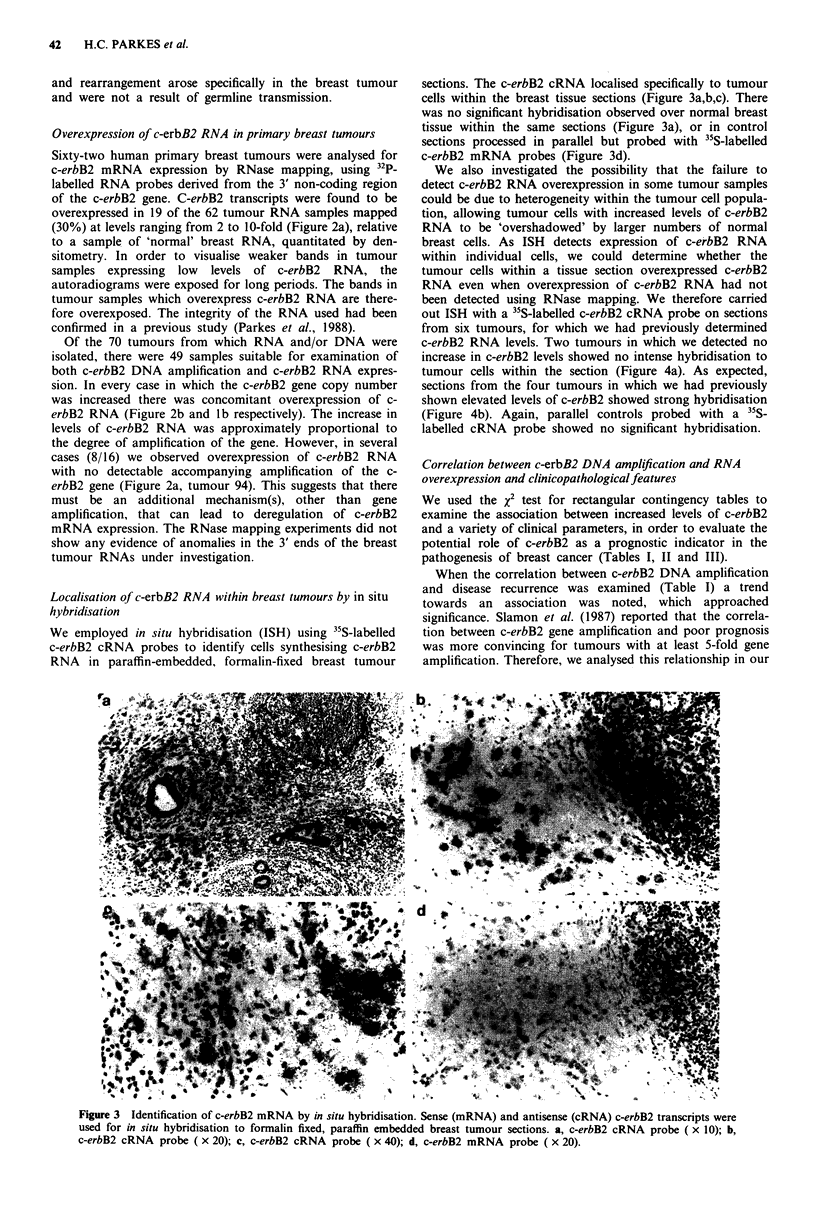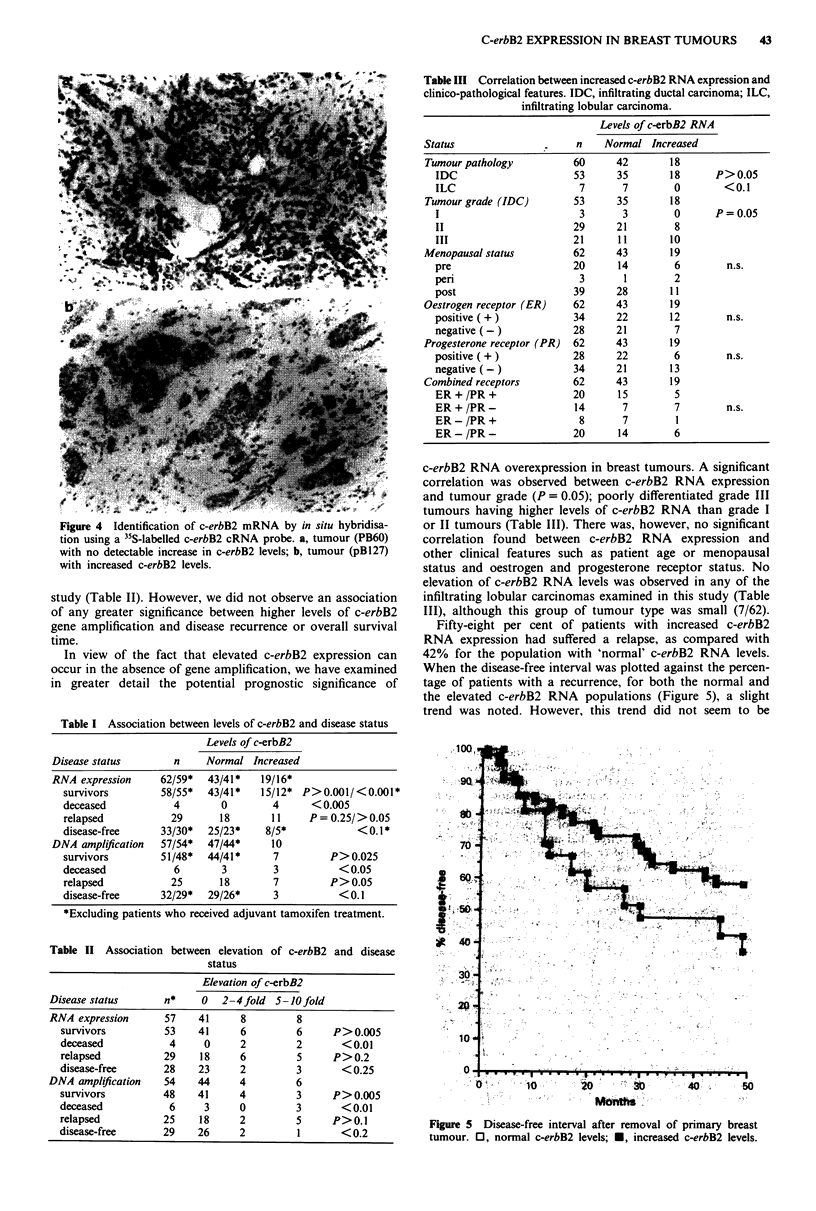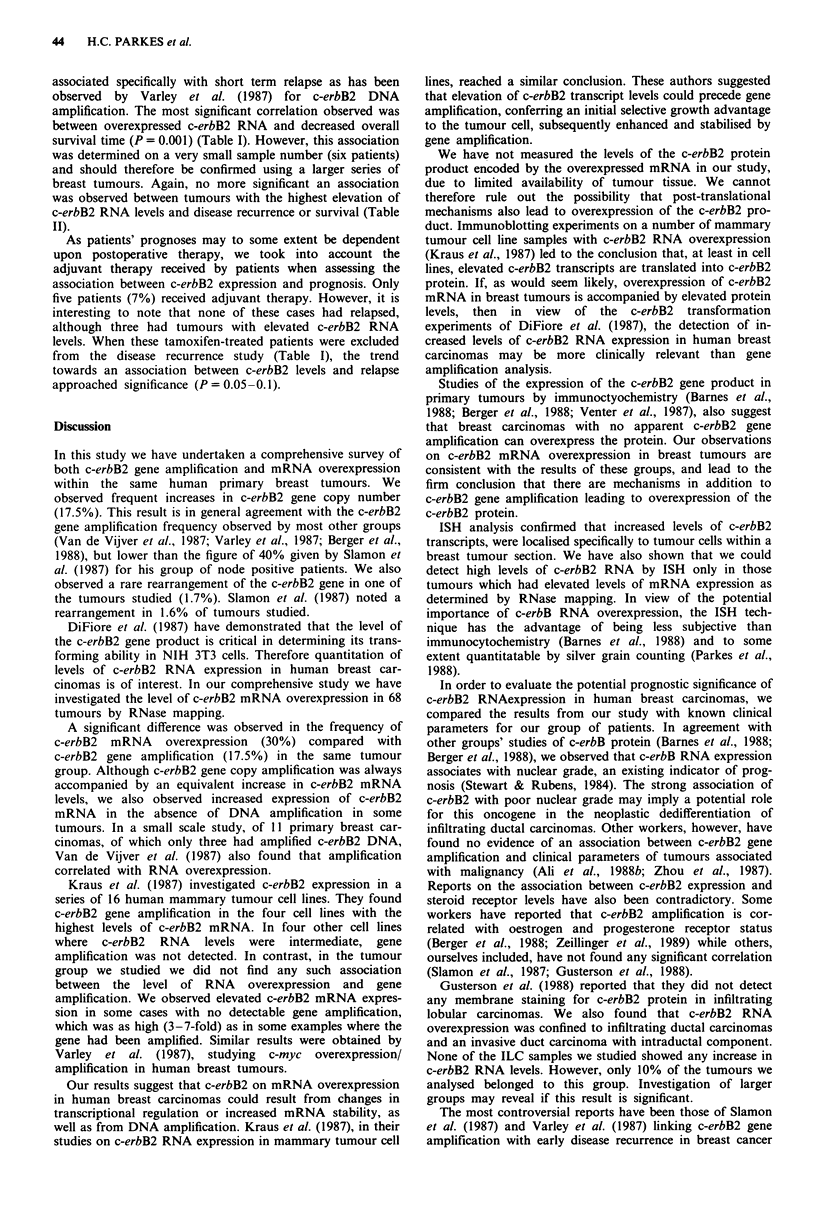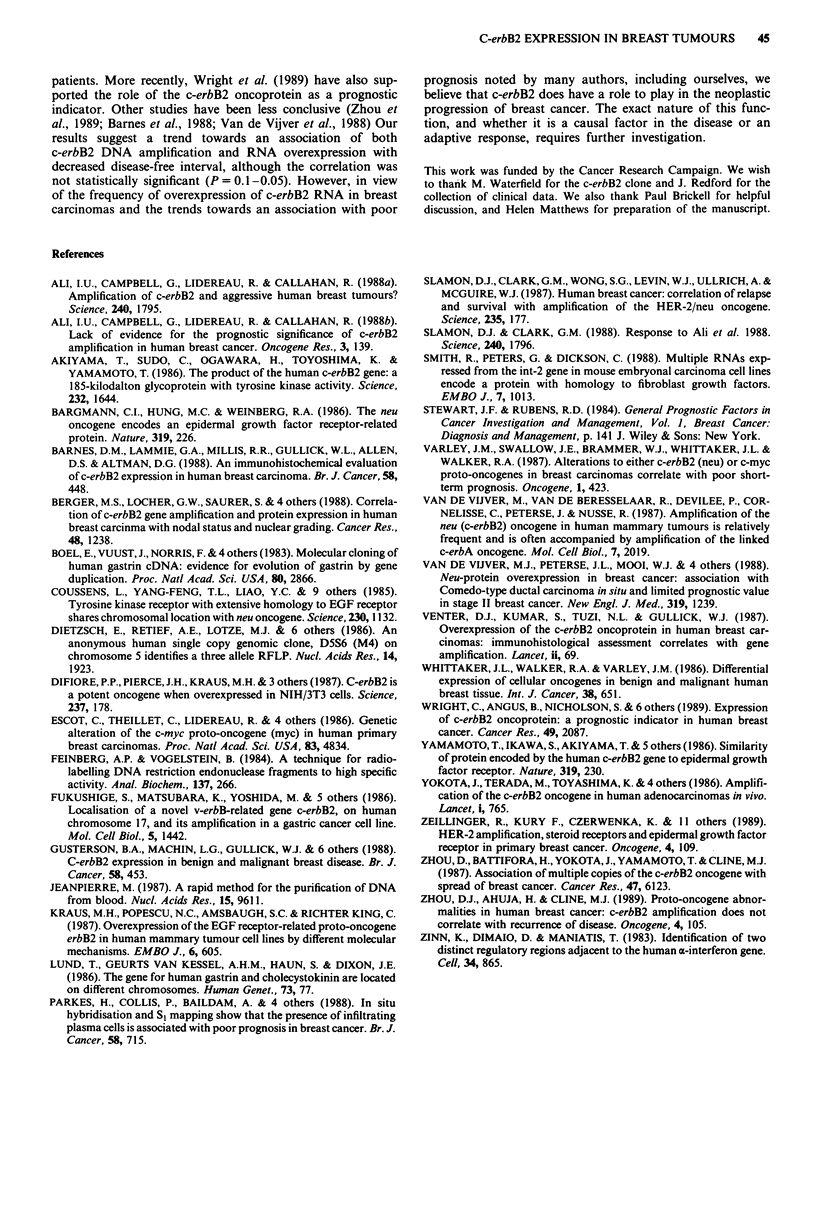# C-erbB2 mRNA expression in human breast tumours: comparison with c-erbB2 DNA amplification and correlation with prognosis.

**DOI:** 10.1038/bjc.1990.9

**Published:** 1990-01

**Authors:** H. C. Parkes, K. Lillycrop, A. Howell, R. K. Craig

**Affiliations:** Department of Biochemistry, University College, London, UK.

## Abstract

**Images:**


*Br. J. Cancer* (1990), **61**, 39–45 © Macmillan Press Ltd., 1990

### C-*erb*B2 mRNA expression in human breast tumours: comparison with c-*erb*B2 DNA amplification and correlation with prognosis

H.C. Parkes[1], K. Lillycrop[1], A. Howell[2] & R.K. Craig[1,*]

[1]*The Cancer Research Campaign Endocrine Tumour Molecular Biology Group, The Medical Molecular Biology Unit, Department of Biochemistry, University College and Middlesex School of Medicine, London WIP 6DP;* [2]*Cancer Research Campaign Department of Medical Oncology, Christie Hospital and Holt Radium Institute, Manchester M20 9BX, UK.*

**Summary** In this study, we have investigated the expression of the proto-oncogene c-*erb*B2 in a total of 70 human primary breast tumours. In agreement with other workers, we observed c-*erb*B2 gene amplification in 17.5% of the tumours studied. In addition, we carried out a comprehensive analysis of c-*erb*B2 mRNA expression in the tumours using RNase mapping and *in situ* hybridisation techniques. Our results indicated a more frequent (30%) overexpression of c-*erb*B2 mRNA, which was associated only with breast carcinomas of a ductal origin. Furthermore, analysis of the c-*erb*B2 mRNA gene locus in the same tumours demonstrated that enhanced c-*erb*B2 expression could occur in the presence or absence of gene amplification, suggesting that additional molecular mechanisms may result in overexpression of c-*erb*B2 mRNA in human mammary tumours. *In situ* hybridisation showed that elevated levels of c-*erb*B2 mRNA were specific to malignant cells within the breast tumour. Analysis of the association between c-*erb*B2 mRNA overexpression and clinico-pathological factors revealed a significant correlation with poor tumour grade, but not with steroid receptor status or patient menopausal status. No significant correlation was observed between overexpression of c-*erb*B2 mRNA and early disease recurrence in our group of patients, although there was a definite trend towards poorer prognosis.

Recent efforts in cancer research have been directed at finding molecular markers of potential predictive value in tumour prognosis. There is increasing evidence to link the activation of cellular proto-oncogenes with the initiation or progression of particular human malignancies. In human breast carcinoma, several such genes have been found to be amplified or rearranged, including c-*myc* and c-*ras* (Whittaker *et al.*, 1986; Escot *et al.*, 1986), although the role of these proto-oncogenes in the progression of the disease is, as yet, unclear.

There is at present much controversy surrounding the potential role of the human proto-oncogene c-*erb*B2 (also known as *neu* or *HER2*) in breast cancer prognosis (Ali *et al.*, 1988*a*; Slamon & Clark, 1988). C-*erb*B2 is related to, but distinct from, the c-*erb*B1/EGF receptor gene (Bargmann *et al.*, 1986; Yamamoto *et al.*, 1986) and maps to band q21 on chromosone 17 (Fukushige *et al.*, 1986; Coussens *et al.*, 1985). It encodes a normal cellular glycoprotein showing structural features of a growth factor receptor, including a transmembrane region and a tyrosine kinase domain (Akiyama *et al.*, 1986), although its ligand has not yet been identified. Many tissues express c-*erb*B2 transcripts. However, observations that amplifications of the c-*erb*B2 gene is limited to carcinomas of glandular epithelial origin (Yokota *et al.*, 1986) have led to the suggestion that the c-*erb*B2 gene encodes a growth factor receptor associated with glandular epithelium (Zhou *et al.*, 1987).

The c-*erb*B2 gene is amplified relatively frequently in human primary breast carcinomas, although the frequency and level of amplification observed in different studies varies widely, in the range 10–40% (Slamon *et al.*, 1987; Van de Vijver *et al.*, 1987; Venter *et al.*, 1987; Ali *et al.*, 1988*b*; Berger *et al.*, 1988). Clinical interest in the c-*erb*B2 gene with respect to breast cancer prognosis has been stimulated by the reports of Slamon *et al.* (1987) and Varley *et al.* (1987) that amplification of the c-*erb*B2 gene is a significant predictor of decreased survival time and rapid relapse in patients. Slamon *et al.* (1987) reported that c-*erb*B2 amplification had greater prognostic significance than most currently used indicators in lymph node-positive disease. However, recent primary breast tumour DNA studies by other groups (Zhou *et al.*, 1987, 1989; Ali *et al.*, 1988*a,b*) have failed to confirm the prognostic significance of this finding. Immunocytochemical investigations on the c-*erb*B2 protein in breast carcinomas by some groups have not established a positive correlation between the expression of c-*erb*B2 and tumour recurrence (Barnes *et al.*, 1988; Van de Vijver *et al.*, 1988). However, Wright *et al.* (1989) have reported that expression of the oncoprotein is an important prognostic indicator.

We have investigated the amplification of the c-*erb*B2 gene in a series of clinically well-defined primary breast tumours, and have examined the levels of expression of c-*erb*B2 mRNA within these tumours by RNase mapping. The degrees of c-*erb*B2 DNA amplification and mRNA overexpression in the tumours have been correlated with clinical parameters of prognosis in order to evaluate whether they are significant predictors of relapse or survival time in breast cancer. Furthermore, since the conflicting results between different tumour studies may be attributed to heterogeneity of the tumour cell population, we have investigated the distribution of c-*erb*B2 mRNA within the breast tumour tissue by *in situ* hybridisation. This allows an assessment of the levels of c-*erb*B2 mRNA in individual normal and malignant cells within the tumour.

### Materials and methods

### *Patients and pathological material*

Material for this study was obtained from 70 patients presenting with primary breast carcinomas at the Christie Hospital and Holt Radium Institute, Manchester, between 1984 and 1986. The majority of these patients (95%) were treated, either by simple mastectomy (52%) or by excision biopsy (43%). The age range of the patients was 33–88, with a median of 60. Tumours excised from these patients included 60 infiltrating ductal carcinomas, seven infiltrating lobular carcinomas, one of mixed type, one invasive duct carcinoma with a predominant intraductal component and one of unknown pathology. Clinical follow-up data, to the time of this study, were available for all of the patients. Of the 70 cases, 54% received no postoperative therapy, 39% received radiotherapy and 7% had adjuvant tamoxifen treatment. Reduction mammoplasty tissue was used as a source of normal breast tissue.

*Present address: Department of Biotechnology, ICI, Alderley Park, Macclesfield, Cheshire SK10 4TG, UK.
Correspondence: H.C. Parkes.
Received 2 May 1989; and in revised form 22 August 1989.

Tumour samples for RNA/DNA extraction were frozen in liquid nitrogen immediately after excision. For *in situ* hybridisation studies, formalin fixed tissue from the same primary breast tumours was processed and embedded in paraffin wax in routine pathology laboratories, without special precautions to prevent RNA degradation. It was then stored at ambient temperatures.

*RNA and DNA extraction*

Tumour tissues DNA and RNA were extracted from the same tumour sample after centrifugation of the homogenised tumour tissue through a CsCl cushion. Total RNA was isolated as described by Parkes *et al.* (1988). The DNA was isolated by removing the interface between the CsCl cushion and the supernatant. This fraction was then diluted to 10 ml with 10mM Tris-HCl (pH 7.5)/10 mM EDTA, and extracted twice with phenol/chloroform/iso amyl alcohol (24:24:1 by volume) and twice with chloroform before ethanol precipitation. Sufficient DNA for further analysis was obtained from 59 out of the 70 tumours, while 62 of the tumour samples yielded RNA of suitable quality for RNase mapping experiments.

DNA was extracted from the peripheral blood lymphocytes of breast tumour patients by the method of Jeanpierre (1987).

*Probes*

*C-*erb*B2* The plasmid pSV2-*erb*B2, which contains a full length c-*erb*B2 cDNA clone in the vector pSV2 (Yamamoto *et al.*, 1986) was digested to completion with either *Kpn* I/*Hind* III or *Taq* I/*Hind* III to release 1234 bp and 656 bp fragments, respectively, from the 3′ region of the c-*erb*B2 gene. These fragments, which have only limited homology to the c-*erb*B1/EGF receptor gene, were subcloned into pGem 4 Blue and pGem 3 Blue respectively.

For Southern blot analysis of c-*erb*B2 the *Kpn* I/*Hind* III fragment was excised from the pGem 4 Blue construct and radiolabelled with $\alpha$-$^{32}$P dCTP (3,000 Ci mmol$^{-1}$) by the random primer method of Feinberg and Vogelstein (1984).

For RNase mapping and *in situ* hybridisation studies, c-*erb*B2 RNA probes were made from the pGem 3 Blue/c-*erb*B2 construct, containing the *Taq* I/*Hind* III c-*erb*B2 fragment. The plasmid was linearised with either *Hind* III or *Eco* RI and transcribed with T7 or Sp6 RNA polymerase, respectively, to produce sense (mRNA) or anti-sense (cRNA) transcripts.

*Human growth hormone (hGH)* An 816 bp *Pst* I cDNA clone (L. Hall, unpublished).

*Gastrin* A 549 bp *Pst* I cDNA clone (Boel *et al.*, 1983).

*D5S6* A human genomic clone containing a 7.6 kb insert representing a single copy sequence from chromosome 5, cloned in the bacteriophage vector L47·1. D5S6 identifies three allelic RFLPs upon hybridisation to *Bam* HI digested genomic DNA (Dietzch *et al.*, 1986).

*DNA analysis*

Five micrograms of DNA were digested with *Eco* RI or *Bam* HI and electrophoresed through a 0.8% agarose gel. The DNA was blotted on to Hybond-N membrane (Amersham International plc; Amersham, UK) and cross-linked to the membrane by UV-illumination as described by the manufacturer. The membrane was hybridised at 65°C overnight with $10^6$ c.p.m. ml$^{-1}$ of radiolabelled probe in 6 × SSC, 5 × Denhardt's solution, 0.5% SDS and 500 µg ml$^{-1}$ of denatured sonicated salmon sperm DNA. The blot was then washed to high stringency (0.5 × SSC at 65°C) and autoradiographed for 1–3 days at −70°C using Kodak X-Omat XAR5 film. Blots were stripped of probe by submerging in boiling water for 5 min and were pre-hybridised and re-hybridised, using a second labelled probe, as before.

*Preparation of RNA probes*

RNA probes radiolabelled with $\alpha$-$^{32}$P CTP (800 Ci mmol$^{-1}$) were synthesised as follows. The pGem Blue 3/c-*erb*B2 construct was linearised with *Eco* RI and transcribed with Sp6 RNA polymerase (Promega) according to the manufacturer's instructions. DNase I (BCL) was added to digest the DNA template. The radiolabelled transcripts were then purified by electrophoresis on a 6% polyacrylamide-urea gel (Smith *et al.*, 1988). Labelled RNA species of the correct size were identified by brief autoradiography, eluted from the gel by shaking overnight in 0.5M ammonium acetate, 10mM magnesium acetate, 1mM EDTA and 0.1% SDS and were then ethanol precipitated with 10 µg of carrier tRNA.

*RNase protection analyses*

Approximately 1 × $10^5$ c.p.m. of purified RNA probe were coprecipitated with 5 µg of tumour total RNA and resuspended in 30 µl of hybridisation buffer (80% formamide, 50 mM PIPES pH 6.7, 400 mM NaCl, 1 mM EDTA) as described by Zinn *et al.* (1983). After heating to 85°C for 20 min, the samples were incubated at 56°C overnight. Three hundred microlitres of RNase digestion buffer (0.3 M NaCl, 10 mM Tris-HCl pH 7.5, 5 mM EDTA, 20 µg ml$^{-1}$ RNase A, 1 µg ml$^{-1}$ RNase T1) was added to each tube and samples were incubated for 30 min at 30°C. The RNase-resistant duplexes were deproteinised by addition of 20 µl of 10% SDS and 50 µg ml$^{-1}$ proteinase K, followed by incubation at 37°C for 15 min, and extraction with phenol/choloroform/isoamyl alcohol (24:24:1 by volume). Residual RNA fragments were then concentrated by ethanol precipitation in the presence of 5 µg tRNA, washed in 70% ethanol and resolved by electrophoresis under denaturing conditions on a 6% polyacrylamide-urea gel.

In situ *hybridisation*

This was carried out essentially as described by Parkes *et al.* (1988), with the following modifications. The c-*erb*B2 RNA transcripts were radiolabelled with $\alpha$-$^{35}$S CTP (1,350 Ci mmol$^{-1}$). The hybridisation was then performed in the presence of 10 mM DTT and the subsequent washes in the presence of 5 mM DTT.

### Results

*C-*erb*B2 gene amplification in primary breast tumours*

DNA isolated from 57 primary breast tumours was analysed with a c-*erb*B2 cDNA probe. Amplification of the human c-*erb*B2 gene was observed in 10 cases (17.5%), all of which were infiltrating ductal carcinomas. The degree of amplification was estimated both by performing serial dilutions of the DNA samples and by laser scanning densitometry.

Figures 1 and 2b show the results obtained from Southern blotting analysis of 16 of the tumour DNA samples. Moderate amplification (2–3-fold) of the 10 kb c-*erb*B2 fragment was seen in tumours 95 and 78, while a higher level of amplification was observed in tumours 40, 64 and 80. The latter tumour had, in addition, a smaller fragment of 5.6 kb which was highly amplified. Preliminary studies have indicated that this represents rearrangement of the c-*erb*B2 gene, a rare event only observed in this single case out of the 57 tumours analysed. Examples of DNA which did not contain amplified c-*erb*B2 gene copies are also shown (Figures 1 and 2b; tumours 21, 42, 70, 69, 117, 106, 59, 94, 112). Normal human lymphocyte DNA was analysed in parallel on each gel as a control for levels of single copy c-*erb*B2.

In order to control for slight differences in loading of tumour DNA and for DNA degradation, all filters were stripped of the c-*erb*B2 probe and rehybridised with D5S6, a single copy genomic DNA probe from chromosome 5. D5S6 hybridises to DNA fragments of 11 kb, 9 kb and 7.5 kb and

**Figure 1** Southern blot analysis comparing the gene copy number of c-*erb*B2 in breast tumour DNA and in lymphocyte DNA from the same patient. Tumour (T)/lymphocyte (L) DNA pairs were digested with *Bam* HI and hybridised to a $^{32}$P-labelled c-*erb*B2 cDNA probe. Lane C shows lymphocyte DNA from a healthy individual. The size markers used (lane M) were a *Hind* III digest of DNA.

**Figure 2** The analysis of c-*erb*B2 mRNA overexpression and DNA amplification in human primary breast carcinomas. **a**, RNase mapping of c-*erb*B2 RNA in a series of breast tumours. RNase-protected fragments of c-*erb*B2 mRNA (656 bp) are shown. A negative control containing *E.coli* tRNA was also analysed (lane R). Lane P shows undigested hybridisation probe. **b**, Southern blotting analysis of c-*erb*B2 DNA in a series of breast tumours. Tumour DNA was digested with *Bam* HI, blotted and hybridised with a $^{32}$P-radiolabelled c-*erb*B2 cDNA probe. **c**, Rehybridisation of the DNA blot (**b**) with a $^{32}$P-radiolabelled gastrin cDNA probe.

therefore provides a suitable marker for the amount of high molecular weight DNA present in each track. The relative intensity of this signal in each tumour track was used to normalise the autoradiographic signals obtained with the other probes (data not shown).

To assess whether the apparent levels of c-*erb*B2 amplification were due to genuine amplification or due to an increase in the copy number of chromosome 17, on which the c-*erb*B2 gene lies (17q21–q22), the Southern blot filters were rehybridised with two probes from chromosome 17. These were a gastrin cDNA probe (localised to 17q) (Lund *et al.*, 1986) (Figure 2c) and a human growth hormone cDNA probe (17q22–q24) (Barsh *et al.*, 1983; data not shown). These genes were present at single copy numbers in all cases, demonstrating that the c-*erb*B2 gene amplification observed was real and not due to an increase in the ploidy of chromosome 17.

Lymphocyte DNA was available for several of the patients whose tumours exhibited amplification of the c-*erb*B2 gene. Southern blot analysis of these tumour–lymphocyte pairs (Figure 1) showed that the amplifications (tumours 40, 64, 95) and the one rearrangement (tumour 80) observed were present only in the tumour DNA of that patient and not in the lymphocyte DNA, suggesting that these amplifications and rearrangement arose specifically in the breast tumour and were not a result of germline transmission.

*Overexpression of c-*erbB2 *RNA in primary breast tumours*

Sixty-two human primary breast tumours were analysed for c-*erb*B2 mRNA expression by RNase mapping, using $^{32}$P-labelled RNA probes derived from the 3′ non-coding region of the c-*erb*B2 gene. C-*erb*B2 transcripts were found to be overexpressed in 19 of the 62 tumour RNA samples mapped (30%) at levels ranging from 2 to 10-fold (Figure 2a), relative to a sample of 'normal' breast RNA, quantitated by densitometry. In order to visualise weaker bands in tumour samples expressing low levels of c-*erb*B2 RNA, the autoradiograms were exposed for long periods. The bands in tumour samples which overexpress c-*erb*B2 RNA are therefore overexposed. The integrity of the RNA used had been confirmed in a previous study (Parkes *et al.*, 1988).

Of the 70 tumours from which RNA and/or DNA were isolated, there were 49 samples suitable for examination of both c-*erb*B2 DNA amplification and c-*erb*B2 RNA expression. In every case in which the c-*erb*B2 gene copy number was increased there was concomitant overexpression of c-*erb*B2 RNA (Figure 2b and 1b respectively). The increase in levels of c-*erb*B2 RNA was approximately proportional to the degree of amplification of the gene. However, in several cases (8/16) we observed overexpression of c-*erb*B2 RNA with no detectable accompanying amplification of the c-*erb*B2 gene (Figure 2a, tumour 94). This suggests that there must be an additional mechanism(s), other than gene amplification, that can lead to deregulation of c-*erb*B2 mRNA expression. The RNase mapping experiments did not show any evidence of anomalies in the 3′ ends of the breast tumour RNAs under investigation.

*Localisation of c-*erbB2 *RNA within breast tumours by* in situ *hybridisation*

We employed *in situ* hybridisation (ISH) using $^{35}$S-labelled c-*erb*B2 cRNA probes to identify cells synthesising c-*erb*B2 RNA in paraffin-embedded, formalin-fixed breast tumour sections. The c-*erb*B2 cRNA localised specifically to tumour cells within the breast tissue sections (Figure 3a,b,c). There was no significant hybridisation observed over normal breast tissue within the same sections (Figure 3a), or in control sections processed in parallel but probed with $^{35}$S-labelled c-*erb*B2 mRNA probes (Figure 3d).

We also investigated the possibility that the failure to detect c-*erb*B2 RNA overexpression in some tumour samples could be due to heterogeneity within the tumour cell population, allowing tumour cells with increased levels of c-*erb*B2 RNA to be 'overshadowed' by larger numbers of normal breast cells. As ISH detects expression of c-*erb*B2 RNA within individual cells, we could determine whether the tumour cells within a tissue section overexpressed c-*erb*B2 RNA even when overexpression of c-*erb*B2 RNA had not been detected using RNase mapping. We therefore carried out ISH with a $^{35}$S-labelled c-*erb*B2 cRNA probe on sections from six tumours, for which we had previously determined c-*erb*B2 RNA levels. Two tumours in which we detected no increase in c-*erb*B2 levels showed no intense hybridisation to tumour cells within the section (Figure 4a). As expected, sections from the four tumours in which we had previously shown elevated levels of c-*erb*B2 showed strong hybridisation (Figure 4b). Again, parallel controls probed with a $^{35}$S-labelled cRNA probe showed no significant hybridisation.

*Correlation between c-*erbB2 *DNA amplification and RNA overexpression and clinicopathological features*

We used the $\chi^2$ test for rectangular contingency tables to examine the association between increased levels of c-*erb*B2 and a variety of clinical parameters, in order to evaluate the potential role of c-*erb*B2 as a prognostic indicator in the pathogenesis of breast cancer (Tables I, II and III).

When the correlation between c-*erb*B2 DNA amplification and disease recurrence was examined (Table I) a trend towards an association was noted, which approached significance. Slamon *et al.* (1987) reported that the correlation between c-*erb*B2 gene amplification and poor prognosis was more convincing for tumours with at least 5-fold gene amplification. Therefore, we analysed this relationship in our

**Figure 3** Identification of c-*erb*B2 mRNA by *in situ* hybridisation. Sense (mRNA) and antisense (cRNA) c-*erb*B2 transcripts were used for *in situ* hybridisation to formalin fixed, paraffin embedded breast tumour sections. **a**, c-*erb*B2 cRNA probe ( × 10); **b**, c-*erb*B2 cRNA probe ( × 20); **c**, c-*erb*B2 cRNA probe ( × 40); **d**, c-*erb*B2 mRNA probe ( × 20).

**Figure 4** Identification of c-*erb*B2 mRNA by *in situ* hybridisation using a $^{35}$S-labelled c-*erb*B2 cRNA probe. **a**, tumour (PB60) with no detectable increase in c-*erb*B2 levels; **b**, tumour (pB127) with increased c-*erb*B2 levels.

study (Table II). However, we did not observe an association of any greater significance between higher levels of c-*erb*B2 gene amplification and disease recurrence or overall survival time.

In view of the fact that elevated c-*erb*B2 expression can occur in the absence of gene amplification, we have examined in greater detail the potential prognostic significance of

**Table I** Association between levels of c-*erb*B2 and disease status

| Disease status | n | *Levels of* c-erb*B2* Normal | Increased | |
|---|---|---|---|---|
| *RNA expression* | 62/59* | 43/41* | 19/16* | |
| survivors | 58/55* | 43/41* | 15/12* | $P>0.001/<0.001$* |
| deceased | 4 | 0 | 4 | $<0.005$ |
| relapsed | 29 | 18 | 11 | $P=0.25/>0.05$ |
| disease-free | 33/30* | 25/23* | 8/5* | $<0.1$* |
| *DNA amplification* | 57/54* | 47/44* | 10 | |
| survivors | 51/48* | 44/41* | 7 | $P>0.025$ |
| deceased | 6 | 3 | 3 | $<0.05$ |
| relapsed | 25 | 18 | 7 | $P>0.05$ |
| disease-free | 32/29* | 29/26* | 3 | $<0.1$ |

*Excluding patients who received adjuvant tamoxifen treatment.

**Table II** Association between elevation of c-*erb*B2 and disease status

| Disease status | n* | *Elevation of* c-erb*B2* 0 | 2–4 fold | 5–10 fold | |
|---|---|---|---|---|---|
| *RNA expression* | 57 | 41 | 8 | 8 | |
| survivors | 53 | 41 | 6 | 6 | $P>0.005$ |
| deceased | 4 | 0 | 2 | 2 | $<0.01$ |
| relapsed | 29 | 18 | 6 | 5 | $P>0.2$ |
| disease-free | 28 | 23 | 2 | 3 | $<0.25$ |
| *DNA amplification* | 54 | 44 | 4 | 6 | |
| survivors | 48 | 41 | 4 | 3 | $P>0.005$ |
| deceased | 6 | 3 | 0 | 3 | $<0.01$ |
| relapsed | 25 | 18 | 2 | 5 | $P>0.1$ |
| disease-free | 29 | 26 | 2 | 1 | $<0.2$ |

**Table III** Correlation between increased c-*erb*B2 RNA expression and clinico-pathological features. IDC, infiltrating ductal carcinoma; ILC, infiltrating lobular carcinoma.

| Status | n | *Levels of* c-erb*B2 RNA* Normal | Increased | |
|---|---|---|---|---|
| *Tumour pathology* | 60 | 42 | 18 | |
| IDC | 53 | 35 | 18 | $P>0.05$ |
| ILC | 7 | 7 | 0 | $<0.1$ |
| *Tumour grade (IDC)* | 53 | 35 | 18 | |
| I | 3 | 3 | 0 | $P=0.05$ |
| II | 29 | 21 | 8 | |
| III | 21 | 11 | 10 | |
| *Menopausal status* | 62 | 43 | 19 | |
| pre | 20 | 14 | 6 | n.s. |
| peri | 3 | 1 | 2 | |
| post | 39 | 28 | 11 | |
| *Oestrogen receptor (ER)* | 62 | 43 | 19 | |
| positive (+) | 34 | 22 | 12 | n.s. |
| negative (−) | 28 | 21 | 7 | |
| *Progesterone receptor (PR)* | 62 | 43 | 19 | |
| positive (+) | 28 | 22 | 6 | n.s. |
| negative (−) | 34 | 21 | 13 | |
| *Combined receptors* | 62 | 43 | 19 | |
| ER+/PR+ | 20 | 15 | 5 | |
| ER+/PR− | 14 | 7 | 7 | n.s. |
| ER−/PR+ | 8 | 7 | 1 | |
| ER−/PR− | 20 | 14 | 6 | |

c-*erb*B2 RNA overexpression in breast tumours. A significant correlation was observed between c-*erb*B2 RNA expression and tumour grade ($P=0.05$); poorly differentiated grade III tumours having higher levels of c-*erb*B2 RNA than grade I or II tumours (Table III). There was, however, no significant correlation found between c-*erb*B2 RNA expression and other clinical features such as patient age or menopausal status and oestrogen and progesterone receptor status. No elevation of c-*erb*B2 RNA levels was observed in any of the infiltrating lobular carcinomas examined in this study (Table III), although this group of tumour type was small (7/62).

Fifty-eight per cent of patients with increased c-*erb*B2 RNA expression had suffered a relapse, as compared with 42% for the population with 'normal' c-*erb*B2 RNA levels. When the disease-free interval was plotted against the percentage of patients with a recurrence, for both the normal and the elevated c-*erb*B2 RNA populations (Figure 5), a slight trend was noted. However, this trend did not seem to be

**Figure 5** Disease-free interval after removal of primary breast tumour. □, normal c-*erb*B2 levels; ■, increased c-*erb*B2 levels.

associated specifically with short term relapse as has been observed by Varley *et al.* (1987) for c-*erb*B2 DNA amplification. The most significant correlation observed was between overexpressed c-*erb*B2 RNA and decreased overall survival time ($P = 0.001$) (Table I). However, this association was determined on a very small sample number (six patients) and should therefore be confirmed using a larger series of breast tumours. Again, no more significant an association was observed between tumours with the highest elevation of c-*erb*B2 RNA levels and disease recurrence or survival (Table II).

As patients' prognoses may to some extent be dependent upon postoperative therapy, we took into account the adjuvant therapy received by patients when assessing the association between c-*erb*B2 expression and prognosis. Only five patients (7%) received adjuvant therapy. However, it is interesting to note that none of these cases had relapsed, although three had tumours with elevated c-*erb*B2 RNA levels. When these tamoxifen-treated patients were excluded from the disease recurrence study (Table I), the trend towards an association between c-*erb*B2 levels and relapse approached significance ($P = 0.05-0.1$).

### Discussion

In this study we have undertaken a comprehensive survey of both c-*erb*B2 gene amplification and mRNA overexpression within the same human primary breast tumours. We observed frequent increases in c-*erb*B2 gene copy number (17.5%). This result is in general agreement with the c-*erb*B2 gene amplification frequency observed by most other groups (Van de Vijver *et al.*, 1987; Varley *et al.*, 1987; Berger *et al.*, 1988), but lower than the figure of 40% given by Slamon *et al.* (1987) for his group of node positive patients. We also observed a rare rearrangement of the c-*erb*B2 gene in one of the tumours studied (1.7%). Slamon *et al.* (1987) noted a rearrangement in 1.6% of tumours studied.

DiFiore *et al.* (1987) have demonstrated that the level of the c-*erb*B2 gene product is critical in determining its transforming ability in NIH 3T3 cells. Therefore quantitation of levels of c-*erb*B2 RNA expression in human breast carcinomas is of interest. In our comprehensive study we have investigated the level of c-*erb*B2 mRNA overexpression in 68 tumours by RNase mapping.

A significant difference was observed in the frequency of c-*erb*B2 mRNA overexpression (30%) compared with c-*erb*B2 gene amplification (17.5%) in the same tumour group. Although c-*erb*B2 gene copy amplification was always accompanied by an equivalent increase in c-*erb*B2 mRNA levels, we also observed increased expression of c-*erb*B2 mRNA in the absence of DNA amplification in some tumours. In a small scale study, of 11 primary breast carcinomas, of which only three had amplified c-*erb*B2 DNA, Van de Vijver *et al.* (1987) also found that amplification correlated with RNA overexpression.

Kraus *et al.* (1987) investigated c-*erb*B2 expression in a series of 16 human mammary tumour cell lines. They found c-*erb*B2 gene amplification in the four cell lines with the highest levels of c-*erb*B2 mRNA. In four other cell lines where c-*erb*B2 RNA levels were intermediate, gene amplification was not detected. In contrast, in the tumour group we studied we did not find any such association between the level of RNA overexpression and gene amplification. We observed elevated c-*erb*B2 mRNA expression in some cases with no detectable gene amplification, which was as high (3–7-fold) as in some examples where the gene had been amplified. Similar results were obtained by Varley *et al.* (1987), studying c-*myc* overexpression/amplification in human breast tumours.

Our results suggest that c-*erb*B2 on mRNA overexpression in human breast carcinomas could result from changes in transcriptional regulation or increased mRNA stability, as well as from DNA amplification. Kraus *et al.* (1987), in their studies on c-*erb*B2 RNA expression in mammary tumour cell lines, reached a similar conclusion. These authors suggested that elevation of c-*erb*B2 transcript levels could precede gene amplification, conferring an initial selective growth advantage to the tumour cell, subsequently enhanced and stabilised by gene amplification.

We have not measured the levels of the c-*erb*B2 protein product encoded by the overexpressed mRNA in our study, due to limited availability of tumour tissue. We cannot therefore rule out the possibility that post-translational mechanisms also lead to overexpression of the c-*erb*B2 product. Immunoblotting experiments on a number of mammary tumour cell line samples with c-*erb*B2 RNA overexpression (Kraus *et al.*, 1987) led to the conclusion that, at least in cell lines, elevated c-*erb*B2 transcripts are translated into c-*erb*B2 protein. If, as would seem likely, overexpression of c-*erb*B2 mRNA in breast tumours is accompanied by elevated protein levels, then in view of the c-*erb*B2 transformation experiments of DiFiore *et al.* (1987), the detection of increased levels of c-*erb*B2 RNA expression in human breast carcinomas may be more clinically relevant than gene amplification analysis.

Studies of the expression of the c-*erb*B2 gene product in primary tumours by immunoctyochemistry (Barnes *et al.*, 1988; Berger *et al.*, 1988; Venter *et al.*, 1987), also suggest that breast carcinomas with no apparent c-*erb*B2 gene amplification can overexpress the protein. Our observations on c-*erb*B2 mRNA overexpression in breast tumours are consistent with the results of these groups, and lead to the firm conclusion that there are mechanisms in addition to c-*erb*B2 gene amplification leading to overexpression of the c-*erb*B2 protein.

ISH analysis confirmed that increased levels of c-*erb*B2 transcripts, were localised specifically to tumour cells within a breast tumour section. We have also shown that we could detect high levels of c-*erb*B2 RNA by ISH only in those tumours which had elevated levels of mRNA expression as determined by RNase mapping. In view of the potential importance of c-*erb*B RNA overexpression, the ISH technique has the advantage of being less subjective than immunocytochemistry (Barnes *et al.*, 1988) and to some extent quantitatable by silver grain counting (Parkes *et al.*, 1988).

In order to evaluate the potential prognostic significance of c-*erb*B2 RNAexpression in human breast carcinomas, we compared the results from our study with known clinical parameters for our group of patients. In agreement with other groups' studies of c-*erb*B protein (Barnes *et al.*, 1988; Berger *et al.*, 1988), we observed that c-*erb*B RNA expression associates with nuclear grade, an existing indicator of prognosis (Stewart & Rubens, 1984). The strong association of c-*erb*B2 with poor nuclear grade may imply a potential role for this oncogene in the neoplastic dedifferentiation of infiltrating ductal carcinomas. Other workers, however, have found no evidence of an association between c-*erb*B2 gene amplification and clinical parameters of tumours associated with malignancy (Ali *et al.*, 1988*b*; Zhou *et al.*, 1987). Reports on the association between c-*erb*B2 expression and steroid receptor levels have also been contradictory. Some workers have reported that c-*erb*B2 amplification is correlated with oestrogen and progesterone receptor status (Berger *et al.*, 1988; Zeillinger *et al.*, 1989) while others, ourselves included, have not found any significant correlation (Slamon *et al.*, 1987; Gusterson *et al.*, 1988).

Gusterson *et al.* (1988) reported that they did not detect any membrane staining for c-*erb*B2 protein in infiltrating lobular carcinomas. We also found that c-*erb*B2 RNA overexpression was confined to infiltrating ductal carcinomas and an invasive duct carcinoma with intraductal component. None of the ILC samples we studied showed any increase in c-*erb*B2 RNA levels. However, only 10% of the tumours we analysed belonged to this group. Investigation of larger groups may reveal if this result is significant.

The most controversial reports have been those of Slamon *et al.* (1987) and Varley *et al.* (1987) linking c-*erb*B2 gene amplification with early disease recurrence in breast cancer patients. More recently, Wright *et al.* (1989) have also supported the role of the c-*erb*B2 oncoprotein as a prognostic indicator. Other studies have been less conclusive (Zhou *et al.*, 1989; Barnes *et al.*, 1988; Van de Vijver *et al.*, 1988) Our results suggest a trend towards an association of both c-*erb*B2 DNA amplification and RNA overexpression with decreased disease-free interval, although the correlation was not statistically significant ($P = 0.1-0.05$). However, in view of the frequency of overexpression of c-*erb*B2 RNA in breast carcinomas and the trends towards an association with poor prognosis noted by many authors, including ourselves, we believe that c-*erb*B2 does have a role to play in the neoplastic progression of breast cancer. The exact nature of this function, and whether it is a causal factor in the disease or an adaptive response, requires further investigation.

This work was funded by the Cancer Research Campaign. We wish to thank M. Waterfield for the c-*erb*B2 clone and J. Redford for the collection of clinical data. We also thank Paul Brickell for helpful discussion, and Helen Matthews for preparation of the manuscript.

### References

ALI, I.U., CAMPBELL, G., LIDEREAU, R. & CALLAHAN, R. (1988*a*). Amplification of c-*erb*B2 and aggressive human breast tumours? *Science*, **240**, 1795.

ALI, I.U., CAMPBELL, G., LIDEREAU, R. & CALLAHAN, R. (1988*b*). Lack of evidence for the prognostic significance of c-*erb*B2 amplification in human breast cancer. *Oncogene Res.*, **3**, 139.

AKIYAMA, T., SUDO, C., OGAWARA, H., TOYOSHIMA, K. & YAMAMOTO, T. (1986). The product of the human c-*erb*B2 gene: a 185-kilodalton glycoprotein with tyrosine kinase activity. *Science*, **232**, 1644.

BARGMANN, C.I., HUNG, M.C. & WEINBERG, R.A. (1986). The *neu* oncogene encodes an epidermal growth factor receptor-related protein. *Nature*, **319**, 226.

BARNES, D.M., LAMMIE, G.A., MILLIS, R.R., GULLICK, W.L., ALLEN, D.S. & ALTMAN, D.G. (1988). An immunohistochemical evaluation of c-*erb*B2 expression in human breast carcinoma. *Br. J. Cancer*, **58**, 448.

BERGER, M.S., LOCHER, G.W., SAURER, S. & 4 others (1988). Correlation of c-*erb*B2 gene amplification and protein expression in human breast carcinma with nodal status and nuclear grading. *Cancer Res.*, **48**, 1238.

BOEL, E., VUUST, J., NORRIS, F. & 4 others (1983). Molecular cloning of human gastrin cDNA: evidence for evolution of gastrin by gene duplication. *Proc. Natl Acad. Sci. USA*, **80**, 2866.

COUSSENS, L., YANG-FENG, T.L., LIAO, Y.C. & 9 others (1985). Tyrosine kinase receptor with extensive homology to EGF receptor shares chromosomal location with *neu* oncogene. *Science*, **230**, 1132.

DIETZSCH, E., RETIEF, A.E., LOTZE, M.J. & 6 others (1986). An anonymous human single copy genomic clone, D5S6 (M4) on chromosome 5 identifies a three allele RFLP. *Nucl. Acids Res.*, **14**, 1923.

DIFIORE, P.P., PIERCE, J.H., KRAUS, M.H. & 3 others (1987). C-*erb*B2 is a potent oncogene when overexpressed in NIH/3T3 cells. *Science*, **237**, 178.

ESCOT, C., THEILLET, C., LIDEREAU, R. & 4 others (1986). Genetic alteration of the c-*myc* proto-oncogene (myc) in human primary breast carcinomas. *Proc. Natl Acad. Sci. USA*, **83**, 4834.

FEINBERG, A.P. & VOGELSTEIN, B. (1984). A technique for radio-labelling DNA restriction endonuclease fragments to high specific activity. *Anal. Biochem.*, **137**, 266.

FUKUSHIGE, S., MATSUBARA, K., YOSHIDA, M. & 5 others (1986). Localisation of a novel v-*erb*B-related gene c-*erb*B2, on human chromosome 17, and its amplification in a gastric cancer cell line. *Mol. Cell Biol.*, **5**, 1442.

GUSTERSON, B.A., MACHIN, L.G., GULLICK, W.J. & 6 others (1988). C-*erb*B2 expression in benign and malignant breast disease. *Br. J. Cancer*, **58**, 453.

JEANPIERRE, M. (1987). A rapid method for the purification of DNA from blood. *Nucl. Acids Res.*, **15**, 9611.

KRAUS, M.H., POPESCU, N.C., AMSBAUGH, S.C. & RICHTER KING, C. (1987). Overexpression of the EGF receptor-related proto-oncogene *erb*B2 in human mammary tumour cell lines by different molecular mechanisms. *EMBO J.*, **6**, 605.

LUND, T., GEURTS VAN KESSEL, A.H.M., HAUN, S. & DIXON, J.E. (1986). The gene for human gastrin and cholecystokinin are located on different chromosomes. *Human Genet.*, **73**, 77.

PARKES, H., COLLIS, P., BAILDAM, A. & 4 others (1988). In situ hybridisation and $S_1$ mapping show that the presence of infiltrating plasma cells is associated with poor prognosis in breast cancer. *Br. J. Cancer*, **58**, 715.

SLAMON, D.J., CLARK, G.M., WONG, S.G., LEVIN, W.J., ULLRICH, A. & MCGUIRE, W.J. (1987). Human breast cancer: correlation of relapse and survival with amplification of the HER-2/neu oncogene. *Science*, **235**, 177.

SLAMON, D.J. & CLARK, G.M. (1988). Response to Ali *et al.* 1988. *Science*, **240**, 1796.

SMITH, R., PETERS, G. & DICKSON, C. (1988). Multiple RNAs expressed from the int-2 gene in mouse embryonal carcinoma cell lines encode a protein with homology to fibroblast growth factors. *EMBO J.*, **7**, 1013.

STEWART, J.F. & RUBENS, R.D. (1984). *General Prognostic Factors in Cancer Investigation and Management, Vol. 1, Breast Cancer: Diagnosis and Management*, p. 141 J. Wiley & Sons: New York.

VARLEY, J.M., SWALLOW, J.E., BRAMMER, W.J., WHITTAKER, J.L. & WALKER, R.A. (1987). Alterations to either c-*erb*B2 (neu) or c-myc proto-oncogenes in breast carcinomas correlate with poor short-term prognosis. *Oncogene*, **1**, 423.

VAN DE VIJVER, M., VAN DE BERESSELAAR, R., DEVILEE, P., CORNELISSE, C., PETERSE, J. & NUSSE, R. (1987). Amplification of the *neu* (c-*erb*B2) oncogene in human mammary tumours is relatively frequent and is often accompanied by amplification of the linked c-*erb*A oncogene. *Mol. Cell Biol.*, **7**, 2019.

VAN DE VIJVER, M.J., PETERSE, J.L., MOOI, W.J. & 4 others (1988). *Neu*-protein overexpression in breast cancer: association with Comedo-type ductal carcinoma *in situ* and limited prognostic value in stage II breast cancer. *New Engl. J. Med.*, **319**, 1239.

VENTER, D.J., KUMAR, S., TUZI, N.L. & GULLICK, W.J. (1987). Overexpression of the c-*erb*B2 oncoprotein in human breast carcinomas: immunohistological assessment correlates with gene amplification. *Lancet*, **ii**, 69.

WHITTAKER, J.L., WALKER, R.A. & VARLEY, J.M. (1986). Differential expression of cellular oncogenes in benign and malignant human breast tissue. *Int. J. Cancer*, **38**, 651.

WRIGHT, C., ANGUS, B., NICHOLSON, S. & 6 others (1989). Expression of c-*erb*B2 oncoprotein: a prognostic indicator in human breast cancer. *Cancer Res.*, **49**, 2087.

YAMAMOTO, T., IKAWA, S., AKIYAMA, T. & 5 others (1986). Similarity of protein encoded by the human c-*erb*B2 gene to epidermal growth factor receptor. *Nature*, **319**, 230.

YOKOTA, J., TERADA, M., TOYASHIMA, K. & 4 others (1986). Amplification of the c-*erb*B2 oncogene in human adenocarcinomas *in vivo*. *Lancet*, **i**, 765.

ZEILLINGER, R., KURY F., CZERWENKA, K. & 11 others (1989). HER-2 amplification, steroid receptors and epidermal growth factor receptor in primary breast cancer. *Oncogene*, **4**, 109.

ZHOU, D., BATTIFORA, H., YOKOTA, J., YAMAMOTO, T. & CLINE, M.J. (1987). Association of multiple copies of the c-*erb*B2 oncogene with spread of breast cancer. *Cancer Res.*, **47**, 6123.

ZHOU, D.J., AHUJA, H. & CLINE, M.J. (1989). Proto-oncogene abnormalities in human breast cancer: c-*erb*B2 amplification does not correlate with recurrence of disease. *Oncogene*, **4**, 105.

ZINN, K., DIMAIO, D. & MANIATIS, T. (1983). Identification of two distinct regulatory regions adjacent to the human α-interferon gene. *Cell*, **34**, 865.